# Suitability of Polymers for 3D-Printing Laboratory Models for Shaking Table Experiments: Discussion and Indications

**DOI:** 10.3390/ma17051172

**Published:** 2024-03-02

**Authors:** Paweł Boroń, Grzegorz Budzik, Joanna Maria Dulińska, Łukasz Przeszłowski, Tadeusz Tatara

**Affiliations:** 1 Faculty of Civil Engineering, Cracow University of Technology, 31-155 Cracow, Poland; jdulinsk@pk.edu.pl (J.M.D.); tadeusz.tatara@pk.edu.pl (T.T.); 2 Faculty of Mechanical Engineering and Aeronautics, Rzeszow University of Technology, 35-959 Rzeszow, Poland; gbudzik@prz.edu.pl (G.B.); lprzeszl@prz.edu.pl (Ł.P.)

**Keywords:** 3D printing, material characteristics, polymers, shaking-table, small-scale model, similarity criteria

## Abstract

This paper presents a comprehensive assessment of the suitability of seven commercially available polymers for crafting laboratory models designed for dynamic shaking-table tests using 3D-printing technology. The objective was to determine whether 3D-printed polymer models are effective for dynamic assessments of structures. The polymers underwent experimental investigations to assess their material properties, i.e., the elastic modulus, the mass density, and the limit of linear-elastic behaviour. The following methodology was applied to obtain the correct values of elasticity moduli and yield points of the polymers: (1) the uniaxial tensile test, (2) the compression test, and (3) the three-point loading test. The filament density was determined as the ratio of sample mass to its volume. The results indicate substantial variations in stiffness, density, and elasticity limits among them. For the similarity analysis, an existing reinforced concrete chimney 120 m high was chosen as a prototype. A geometric similarity scale of 1:120 for a laboratory mock-up was adopted, and a numerical model of the mock-up was created. The similarity scales were calculated for mock-ups made of each filament. Based on these scales, numerical calculations of natural frequencies and dynamic performance under a strong earthquake were carried out for models made of different polymers. Assessment of the polymers’ suitability for laboratory models revealed positive outcomes. The agreement between field experiments, shaking-table tests, and numerical predictions in terms of natural frequencies was observed. Maximum stresses resulting from the earthquake indicated the satisfactory performance of the model below the linear-elastic limit. Despite differences in material properties, the selected polymers were deemed suitable for 3D-printing models for shaking-table tests. However, the discussion raised some important considerations. The upper frequency limit of the shaking-table imposes restrictions on the number of natural frequencies that can be determined. Numerical assessments of natural frequencies are recommended to prevent underestimation and to assess the feasibility of their determination. Additionally, resonance during natural frequency determination may lead to exceeding the linear-elastic limit, affecting filament properties, and making the similarity criteria invalid. Practically, this research contributes insights for planning shaking-table tests, aiding in selecting the most suitable filament and highlighting crucial considerations to ensure reliable and accurate dynamic assessments.

## 1. Introduction

Dynamic experiments on existing structures (prototypes) are frequently substituted with tests conducted on mock-ups of these structures (models) using shaking-tables. Through these tests, it is possible to assess both the dynamic characteristics of laboratory models as well as the behaviour under dynamic loads. Furthermore, by ensuring that the laboratory model adheres to the so-called similarity criteria [1], outcomes derived from the model can be extrapolated to provide insights into the behaviour of the prototype.

Meeting the similarity criteria can be challenging when the laboratory model is made of the same material as the prototype. For this reason, in the laboratory model, the prototype material is replaced by another material that allows the similarity criteria to be met. Currently, a common option is the use of 3D-printed materials, which are becoming more available and cheaper. This method allows the complex geometry of a prototype with relatively thin walls to be replicated in a laboratory model.

The analysis of the reliability and seismic resistance of a prototype building with irregular geometry (rounded walls and vaults) is presented in [2]. The model of the prototype, which is to be entirely made using 3D-printing technology, was also created using 3D concrete printing. During shaking-table tests, the strength of key elements such as walls and roofs was determined. The research confirmed that the real 3D-printed building would meet the design requirements.

The seismic resistance of irregular structures was also studied in works [3,4,5]. The use of 3D printing allowed for a multi-variant analysis of geometric and structural solutions aimed at improving the seismic resistance of objects already at the design stage.

In work [3], the authors presented an analysis of the so-called Quake Columns. This structure is made of cut, fitted stone blocks, connected without mortar. The model was prepared using 3D printing. Its elements were printed and then arranged according to the prototype pattern without mortar. The shaking-table tests showed that the prototype structure is resistant to seismic excitation. A similar technique for building an experimental model was used in the study [4]. The authors analysed the dynamic response of a masonry arch to seismic excitation. The experimental model of the structure was constructed using 3D-printed individual elements. Seismic tests of the model were carried out on a shaking-table, and the results obtained during laboratory experiments were compared with numerical solutions. The seismic research carried out in [5] was aimed at determining the optimal shape of openwork coatings used as vaults. The model of the structure was printed in a 3D printer. Shaking-table tests allowed the authors to assess the seismic resistance of several construction variants.

Three-dimensional printing has also been used in research on underground pipelines. In [6], a dynamic analysis was performed to determine the safety of an underground pipeline during an earthquake. The 3D-printed model of the pipeline coat was buried in the ground. Shaking-table tests allowed for the assessment of the seismic behaviour of the pipeline, considering geotechnical phenomena.

The authors of the work [7] focused on the stability of a slope consisting of rock blocks subjected to seismic accelerations. The slope model was constructed using 3D-printed blocks of various shapes. The conducted shaking-table tests demonstrated which elements of the model (technological solutions or production details) can influence discrepancies between observed and predicted failure modes. In the article [8], the use of 3D printing to calibrate numerical models of damaged objects is presented. The authors performed shaking-table tests on a 3D-printed frame to determine the dynamic characteristics of a previously damaged prototype. Based on the results, it was possible to adjust the material parameters of the numerical model to the actual condition of the tested structure and calibrate the numerical model. One of the latest trends observed is the dynamic analysis of seismic isolators. Several new technical solutions for isolators are presented in the works [9,10,11]. The isolator elements were printed using 3D technology, in which its geometry can be freely modified without significant costs. Moreover, through shaking-table tests, it was possible to monitor the effectiveness of the proposed design changes, which allowed the assessment of the modifications in real time.

From the short review presented above, it is clearly visible that 3D-printed materials have become widely used in structural dynamics. However, it should be remembered that 3D-printing polymers offered by manufacturers may have significantly different purposes, and therefore, they may vary in their mechanical parameters. Due to this reason, some of them probably cannot be applied in shake-table testing.

The main objective of this paper is to recognize a wide range of commercially available polymers used in 3D-printing technology in terms of their suitability for preparing laboratory models for dynamic tests on shaking-tables. In the study, a set of seven polymers, which differ significantly in terms of stiffness, density, elasticity limit, and ease of processing, were examined. Achieving the above objective allows one to answer the question of whether models for shaking-table tests, made of 3D-printed polymers, are a good solution as far as the proper dynamic assessment of structures is concerned. 

This paper’s novelty lies in providing the complex studies that cover the following:

- The experimental investigation of properties for a wide range of commercial polymers that differ significantly in terms of stiffness, density, elasticity limit, and ease of processing;
- The numerical analyses of dynamic properties of the models printed from the selected polymers, as well as the discussion and indications of their suitability for preparing laboratory models used in shaking-table tests in the context of meeting the similarity criteria.

The recognition of the material properties of selected polymers along with numerical studies of their dynamic behaviour during shaking-table tests reveal some pitfalls and provide indications for the use of polymers for 3D-printed mock-ups. To the best of the authors’ knowledge, linking the experimental research on material properties of a wide range of polymers with the assessment of their suitability for preparing 3D-printed models for shaking-table purposes has not yet been addressed in the literature. It makes this work original and unique in the civil engineering area.

## 2. Materials and Methods

### 2.1. Polymers Selected for the Analysis and Their Technological Parameters

In this work, the following seven polymers were selected to assess their suitability for preparing 3D-printed laboratory models for experiments on a shaking-table: (1) polyethene terephthalate (PET-G), (2) PolyJet photopolymer (RDG720), (3) high-impact polystyrene (HIPS), (4) acrylonitrile butadiene styrene copolymer (ABS), (5) bioplastic (polylactic acid) (ULTRA-PLA), (6) Fiberflex (30D), and (7) Fiberflex (40D). The primary reasons for selecting these materials include (1) their biodegradability, (2) ease of processing and production of laboratory models, and (3) wide availability on the market, providing a significant advantage in mock-up production. The selected materials differ in terms of stiffness, density, elasticity limit, and ease of processing. The basic technological parameters used for 3D printing of the samples are presented in Table 1.

### 2.2. Similarity Scales Used in Structural Dynamics

Experimental models, used for shaking-table tests, differ from prototypes in both dimensions and materials. Models must meet similarity criteria [12,13,14] to guarantee that the behaviour of the models during shake-table tests reflects the dynamic behaviour of the prototype. The theory of similarity is defined by the π-Buckingham theorem [15], according to which every physical quantity can be represented by dimensionless variables. The similarity scales take the following form (1):

(1) $$S_{L}=\frac{L_{model}}{L_{prototype}}$$

where

$S_{L}$—the similarity scale for variable *L*;

$L_{model}$—the value of the variable *L* in the model;

$L_{prototype}$—the value of the variable *L* in the prototype.

There are three main types of experimental models: a True Strength Model, an Artificial Mass Model, or an Ignoring Gravity Model. Each type is defined by different similarity criteria and has a different application [16,17]. In this study, the Ignoring Gravity Model (IGM) was implemented. This type of model can only be used when the stresses due to gravity are much smaller than the stresses induced by dynamic loads and when the model operates in an elastic–plastic state [17,18]. The dimension base in the IGM involves the model size (length) *d*, the elasticity modulus *E*, and the material density *ρ*. Similarity scales for the model are therefore determined based on the above three parameters. It should be noted that similarity scales regarding the parameters of the dimension base are determined according to Equation (1), while the remaining similarity scales are determined by appropriately multiplying the base scales. It should be strongly pointed out that the IGMs may be used for linear analyses only. In studies of non-linear phenomena, various errors and inaccuracies may appear if gravitational forces are omitted. The most important similarity scales used in structural dynamics for the IGM are listed in Table 2. 

### 2.3. Procedures for Experimental Determination of Material Properties of the Selected Polymers 

#### 2.3.1. General Assumptions for the Investigations on the Material Properties

A reliable assessment of the suitability of 3D-printed polymers for making experimental models for shaking-table testing should be based on knowledge of the physical and mechanical properties of this material. Especially, material parameters included in the dimensional basis of the model (see Table 2), i.e., the elastic modulus and the mass density, must be precisely determined. It is also necessary to determine the yield point of each filament since the similarity scales are valid only for the linear-elastic behaviour of models.

To guarantee obtaining the correct values of elasticity moduli of the polymers, we decided to use three independent methods: (1) the uniaxial tensile test, (2) the compression test, and (3) the three-point loading test. The average values of the elastic moduli obtained from the three above-mentioned tests were taken for further dynamic analyses. 

All tests served to determine the elasticity moduli and were conducted on the Zwick Roell testing machine on specimens made from all selected polymers. The shapes of the samples used in tensile, compression, and three-point loading tests were chosen based on the Polish Standards recommendation. To prevent the delamination of the samples during the test, the procedure of continuous application of sequential filament layers (no breaks in the process) was executed. 

In 3D-printing technology, samples have a layered structure which results from successive application of a filament. The most common lamination configurations are transverse, longitudinal, and cross-hatched arrangements [19,20]. Almost all specimens were prepared using the cross-hatched pattern of lamination. One could expect that samples made in the different arrangements may exhibit anisotropic features. However, research provided in [19] proved that the modulus of elasticity of the 3D-printed material does not differ significantly (up to 1%) for various directions of lamination. 

For statistical reasons, a series of 6 samples was prepared from each filament. Hence, 42 samples were tested using three methods of testing.

#### 2.3.2. The Experimental Set-Up for the Uniaxial Tensile Test

**Figure 1:**
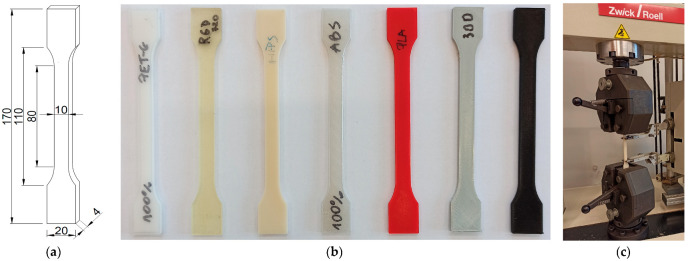
(**a**) The dimensions of the dumbbell-shaped specimen for the uniaxial tensile test (unit: mm); (**b**) the samples made of the selected polymers; (**c**) the specimen mounted in the Zwick Roell machine (https://www.zwickroell.com/; accessed on 28 February 2024) [23].

In the uniaxial tensile tests, the determination of the elastic moduli of polymers was carried out based on the recommendations described in the Polish Standards PN-EN ISO 527-1:2020-01 [21] and PN-EN ISO 527-2:2012 [22]. The dumbbell-shaped specimen dimension, the set of samples, and the mounting of the specimen in the testing machine are shown in Figure 1. 

The distance between the extensometer grips was adjusted to 75 mm. Each sample was subjected to an initial stress of 0.5 MPa, which was 0.05% of the expected value of the elastic modulus. The sample was then stretched at a constant strain rate of 1% per minute. During the test, the change in the distance of the extensometer grips, the displacement of the head, and the value of the tensile force were recorded with a time step of 0.02 s (with a recording frequency of 50 readings per second). The experiment was performed until the specimen deformation reached 0.25%. The value of the elasticity modulus was evaluated according to Formula (2):

(2) $$E=\frac{\sigma_{2}-\sigma_{1}}{\varepsilon_{2}-\varepsilon_{1}}$$

where 

$\sigma_{1}$ and $\sigma_{2}$—stress values under strains $\varepsilon_{1}=0.05\%$ and $\varepsilon_{2}=0.25\%$, respectively.

According to normative recommendations [21], the material’s modulus of elasticity was determined at a temperature within the range of 23 +/− 2 °C.

#### 2.3.3. The Experimental Set-Up for the Compression Test

**Figure 2:**
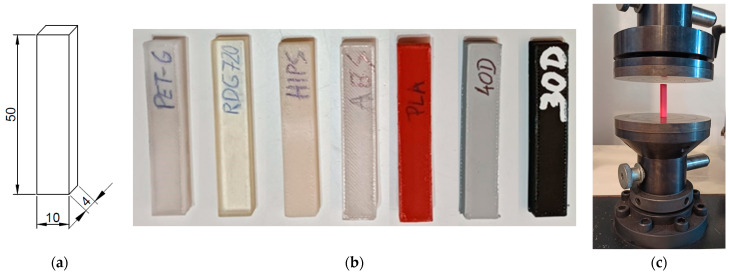
(**a**) The dimensions of the cuboid-shaped specimen for the compression test (unit: mm); (**b**) the samples made of the selected polymers; (**c**) the specimen mounted in the Zwick Roell machine (https://www.zwickroell.com/; accessed on 28 February 2024) [23].

In the compression tests, the determination of the elastic moduli of polymers was performed based on the recommendations described in the Polish Standard PN-EN ISO 604-2006 [24]. The set of samples along with the cuboid-shaped specimen dimension, as well as the mounting of the specimen in the Zwick Roell testing machine, is shown in Figure 2.

During the tests, the samples were placed in the centre of the lower plate of the machine and pressed with the upper plate. Each sample was subjected to axial compression at a constant rate of 2% strain per minute. The experiment was performed until the deformation of the specimen reached 2%. The sample deformation was determined based on the displacements of the upper plate and the compressive force, recorded every 0.02 s. As in the case of the axial tensile tests, the value of the elastic modulus was determined based on Formula (2).

The value of the elasticity modulus during the compression test was determined at a temperature within the range of 23 +/− 2 °C [24].

#### 2.3.4. The Experimental Set-Up for the Three-Point Loading Test

**Figure 3:**
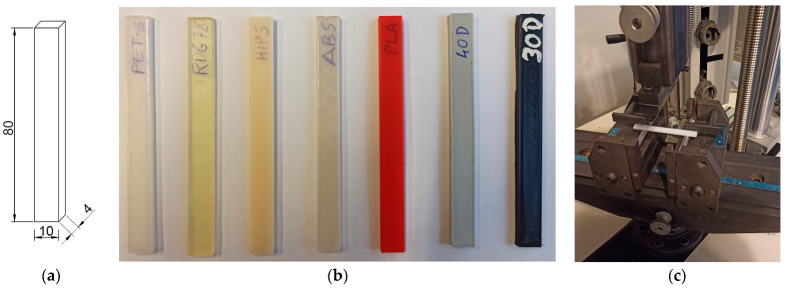
(**a**) The dimensions of the cuboid-shaped specimen for the three-point loading test (unit: mm); (**b**) the samples made of the selected polymers; (**c**) the specimen mounted in the Zwick Roell machine (https://www.zwickroell.com/; accessed on 28 February 2024).

In the three-point loading tests, the evaluation of the elastic moduli of polymers was performed based on the methodology recommended in the Polish Standard PN-EN ISO 178-2019-06 [25]. The set of the cuboid-shape samples with their dimensions and the three-point loading test in the Zwick Roell machine are shown in Figure 3. 

The distance of the external supports was 63 mm. During the three-point loading tests, the bending of a sample was accomplished by applying a load at the mid-span at a constant rate of 1% strain per minute. The vertical displacements of the machine head and the force acting on the sample were recorded every 0.02 s.

The value of the elasticity modulus was evaluated according to Formula (3):

(3) $$E=\frac{\sigma_{f2}-\sigma_{f1}}{\varepsilon_{2}-\varepsilon_{1}}$$

where

$\sigma_{f1}$ and $\sigma_{f2}$—stress values under strains $\varepsilon_{f1}=0.05\%$ and $\varepsilon_{f2}=0.25\%$, respectively.

The stress value $\sigma_{fi}$ is determined based on Formula (4):

(4) $$\sigma_{fi}=\frac{3\cdot F_{i}\cdot L}{2\cdot b\cdot h^{2}}$$

where 

*F_i_*—the bending force acting on a sample;

*L*—the external supports distance; 

*b*—the sample width; 

*h*—the sample height.

The strain values of strain $\varepsilon_{fi}$ for which the stress $\sigma_{fi}$ is calculated from Formula (5):

(5) $$\varepsilon_{fi}=\frac{6\cdot s_{i}\cdot h}{L^{2}}$$

where 

$s_{i}$—the displacement of the head.

The three-point loading tests were conducted at a temperature of 23 +/− 2 °C [25].

#### 2.3.5. Evaluation of the Linear-Elastic Limits of Selected Polymers 

The limit of linear-elastic behaviour of the filament as well as the yield point were also determined, based on the uniaxial tensile test. The stretching of each sample continued until it broke. 

**Figure 4:**
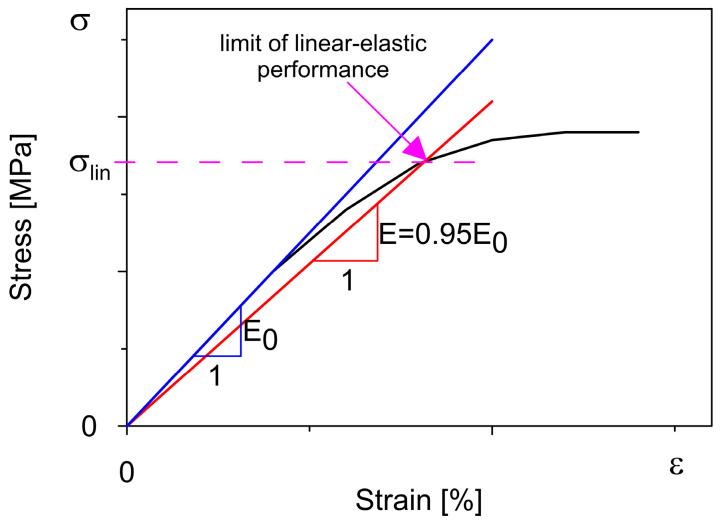
The concept of determining the limit of linear-elastic performance of the filament and the calculation of the secant elasticity modulus (blue line—ideal linear-elastic behaviour with E_0_; red line—ideal linear-elastic behaviour with 0.95E_0_; black line—real behaviour of sample) [26,27].

The determination of the linear-elastic limit of a filament is shown schematically in Figure 4 [26,27]. Materials printed using 3D technology may exhibit slightly non-linear behaviour even at small strains. Therefore, it was assumed that the analysed polymers would work in the linear-elastic range until the secant elasticity modulus *E*, determined for the current value of stress and strain, was not less than 95% of its initial value *E*_0_ (i.e., *E =* 0.95*E*_0_). The initial value of the elasticity modulus *E*_0_ was assessed based on the stress–strain relationship for the strain range 0.05–0.25%.

#### 2.3.6. Determination of the Density of Selected Polymers

The density of the filament was determined as the ratio of sample mass to its volume. This procedure can be considered correct for homogeneous materials only. It is therefore assumed that the sample is made precisely, i.e., it does not contain air voids, delamination, or inclusions. The porosity of the samples, which may result from the presence of holes and a gap between the individual fibres of the laminate, was neglected in the sample volume. Such a simplification was considered acceptable in engineering applications [28].

## 3. Results of Experiments

### 3.1. Experimentally Determined Elastic Moduli of the Selected Polymers

**Figure 5:**
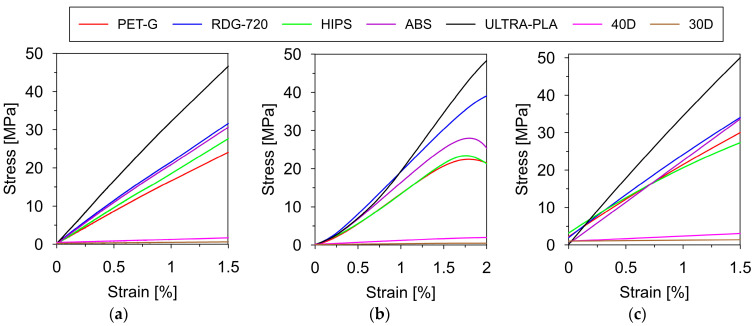
Stress–strain relationships obtained from (**a**) the uniaxial tensile tests; (**b**) the compression tests; (**c**) the three-point loading tests.

The elastic modulus of each filament was determined based on the three independent methods: (1) the uniaxial tensile test, (2) the compression test, and (3) the three-point loading test (see Section 2.3.1). The chosen stress–strain relationships for all polymers obtained from tensile, compression, and bending tests are presented in Figure 5. 

It can be noticed from Figure 5b, that in the initial phase of the tests (at strains less than 0.25%), the stress–strain relationships were non-linear. Linear behaviour of the polymers was detected when the strain values exceeded 0.25%. This phenomenon was observed for each sample, regardless of the filament it was made of. This problem could result from imperfections in the contact surface of the sample with the machine plates. With a small compressive force and small contact area, the results could be distorted. At higher values of compressive force (associated with larger deformations), the imperfections of the contact surface did not affect the results. Considering these explanations, we decided to determine the modulus of elasticity in the range of linear behaviour of the samples. Hence, in the calculations of the elasticity modulus from Formula (2), the values of strains $\varepsilon_{1}=0.25\%$ and $\varepsilon_{2}=1.5\%$ were implemented. Based on the results collected from the tensile, compression, and bending tests, the average elastic moduli of all polymers were calculated. The results are summarized in Table 3.

It can be observed that the values of elastic moduli of the analysed polymers differ significantly. The polymers marked 30D and 40D proved to be the most flexible materials, while the ULTRA-PLA filament turned out to be the stiffest one. Generally, the stiffness of these polymers varied by two orders of magnitude. 

It is also worth emphasizing that the value of the standard deviation for each series of samples was small. This means that the samples were made with similar accuracy and repeatability. Moreover, differences in the values of elastic moduli of the individual filament, obtained with various methods, did not exceed 10%.

### 3.2. Experimentally Detected Linear-Elastic Limits of the Selected Polymers

The linear-elastic limits of the polymers were determined based on the results of the tensile tests. The method of assessing the limits is described in Section 2.3.5. The average values of linear-elastic limits obtained for all polymers are summarized in Table 4.

### 3.3. Experimentally Detected Mass Density of the Selected Polymers

The method of determining the mass density of the filament is described in Section 2.3.6. The average values of mass density obtained for all polymers are shown in Table 5.

## 4. Discussion on the Suitability of the Polymers for 3D-Printed Models for Shaking-Table Tests

### 4.1. A Structure Selected as a Prototype and Its Natural Frequencies

**Figure 6:**
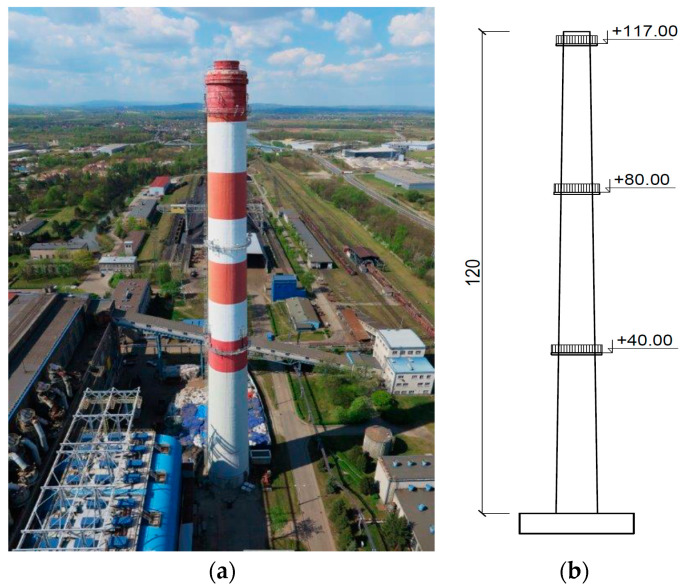
The chimney (the prototype): (**a**) the general view [30], (**b**) the main dimensions (unit: m).

Determining the material properties of the polymers allows for similarity scale calculation. An existing reinforced concrete chimney 120 m high was chosen as a prototype for further consideration [29] (Figure 6). The shaft’s diameter varied from 5.5 to 8.7 m. Its thickness was in the range of 18–50 cm. The thickness of the ceramic lining was 25 cm. The thickness of the carbon deposits, measured in the inspection of the structure, was approximately 5 cm. The elasticity modulus of the shaft was 32 GPa. The mass density of the shaft and the ceramic lining were 2500 and 2400 kg/m^3^, respectively. The mass density of the carbon deposits was 1900 kg/m^3^. 

**Figure 7:**
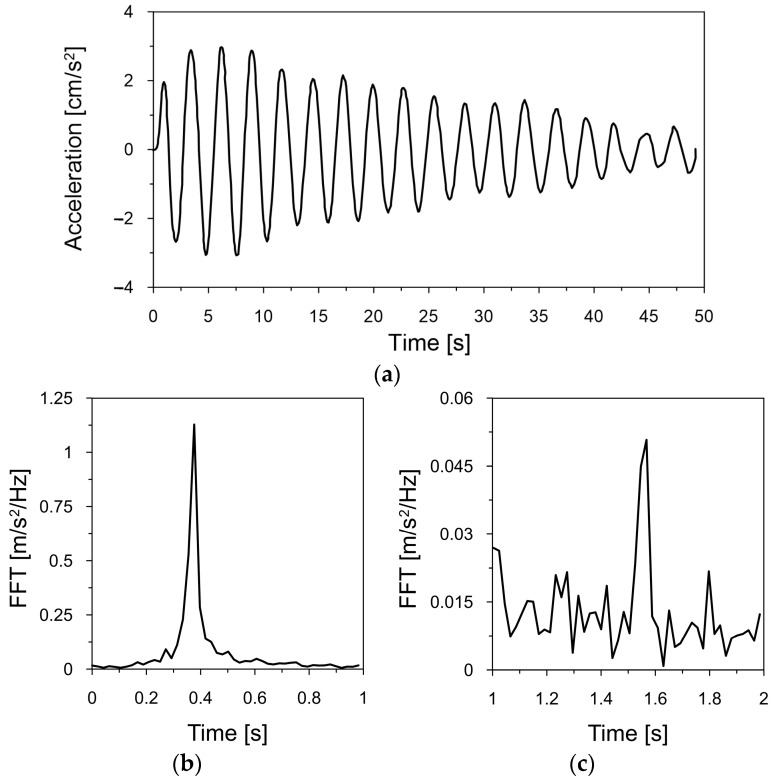
(**a**) The acceleration–time history registered at the top of the chimney; the fragments of the Fourier spectrum served for the estimation of the first (**b**) and the second (**c**) natural frequency [30].

The full description of the chimney, as well as the methods and the results of in situ determination of natural frequencies of the chimney, can be found in [29,30]. The exemplary acceleration–time history of the chimney vibration, executed by people swaying rhythmically at the top gallery of the chimney, is shown in Figure 7a. The fragments of the Fourier spectra, presented in Figure 7b,c, show that the fundamental and the second natural frequency were 0.365 and 1.570 Hz, respectively. 

### 4.2. Similarity Scales for the Selected Polymers

In this work, we decided to adopt a geometric scale of 1:120 for all models, so the height of the models was 1 m. It resulted from the limitations of the shaking-table working space. Two other similarity scales constituting the dimension base, i.e., the elasticity and the density scale, were calculated as a ratio of the elastic modulus of the prototype material (reinforced concrete) to the model material (filament) and the ratio of the prototype material density to the filament density, respectively. The similarity scales calculated for such a dimension base (see Section 2.2) are summarized in Table 6.

### 4.3. The Numerical Model of the Chimney Mock-Up and Its Natural Frequencies

**Figure 8:**
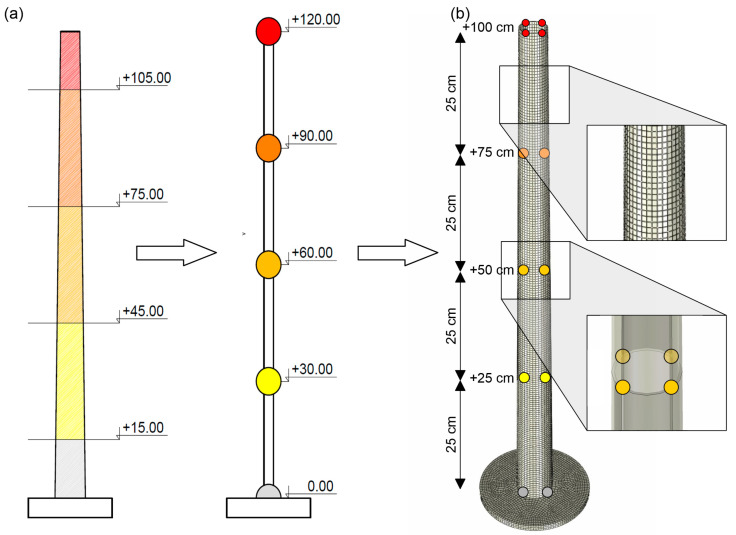
(**a**) The general scheme for additional mass determination (unit: m), (**b**) the arrangement of masses in the numerical model.

The numerical model of the laboratory mock-up, made of filament, was created in Abaqus FEA [31]. The FE model of the mock-up consists of about 14,500 finite elements. The eight-node linear brick elements C3D8R (3 degrees of freedom in node, reduced integration with hourglass control) were used in the numerical model (see Figure 8b). The model of the chimney represented only the concrete chimney shaft. However, the mass resulting from the lining had to be considered as well. Thus, the numerical model was fitted with additional masses to reflect the mass of the lining in the model. This total mass of the lining (1006.5 tons) was divided into five parts, located on the levels of 120, 90, 60, 30, and 0 m above the ground. According to the geometrical scale, the locations of the additional masses in the numerical model were 1.0, 0.75, 0.5, and 0.25 m above ground level (the mass attached to the ground level was not included in the model). The values of additional masses introduced into the numerical model were determined based on the mass similarity scales for the individual polymers (see Table 6). To model the additional masses, lumped mass (point mass) elements were used. The numerical model of the mock-up was fixed (no displacement or rotation). Such boundary conditions allowed for the representation of the chimney mounting during the tests on the shaking-table. The distribution of the ceramic lining mass of the chimney (the prototype) and the arrangement of the additional masses of the laboratory model are summarized in Table 7. The general scheme for additional mass determination and the arrangement of masses in the numerical model is presented in Figure 8.

The first and the second natural frequency of the chimney (the prototype) obtained experimentally were 0.365 and 1.579 Hz, respectively (see Section 4.1). The analyses of the first and the second natural frequencies obtained numerically for the models made of selected polymers are provided in Table 8 and Table 9.

The first natural frequency of the model made of individual filament as well as the first frequency of the chimney (the prototype) calculated based on the similarity scale (scaled frequency) are shown in Table 8, together with the difference between the numerical and the experimental values. The same summary regarding the second natural frequency is provided in Table 9. For such a level of complexity of the problem, the resemblance of the numerical and experimental results may be considered reasonable, since the differences are lower than 1.3 and 4.5% for the first and the second frequency, respectively.

### 4.4. Indications for Determining the Natural Frequencies of the Model

Shaking-tables have upper frequency limits at which stable excitation can be obtained. In the case of large values of frequency similarity scales, the natural frequencies of the model may exceed the upper limit of the shaker excitation frequency. For example, the second natural frequency of the analysed chimney (prototype) was 1.57 Hz, and the frequency similarity scale of the model made of the ULTRA-PLA material was 54.04 (see Table 6). Hence, the excitation frequency of 84.84 Hz must be executed by a shaker. Excitation of such a high frequency is often not possible on typical shaking-tables. 

The natural frequencies of the chimney (the prototype) as well as the frequencies of the models printed with selected polymers are shown in Table 10. The frequencies of the models were obtained using the frequency similarity scales. It is visible from Table 10 which natural frequencies of the models (marked in grey) could not be obtained using a typical shaking-table with a maximum excitation frequency of 80 Hz. It turned out that in the case of the model printed from the ULTRA-PLA filament, it was possible to identify only the first natural frequency of the model. Printing the model with PET-G, RDG 720, HIPS, or ABS filament allowed for identifying two basic frequencies of the mock-up. Finally, the detection of four basic frequencies was possible only for the models printed with the 40D or 30D filament. Therefore, the 40D and 30D polymers have the most beneficial parameters in terms of their suitability for 3D-printing models for shaking-tables and for meeting the frequency similarity criteria or higher natural frequencies. 

In conclusion, the number of model natural frequencies that can be determined is strictly limited by an upper frequency limit of a shaker. It is strongly recommended to assess the natural frequencies of a model numerically to avoid underestimating the fundamental frequencies and making them impossible to excite by a shaker. The above considerations also show that models for shaking-table tests should have the lowest possible values of frequency similarity scales. This fact should be considered when selecting a filament for a model.

### 4.5. Indications for the Linear-Elastic Behaviour of the Model

Similarity scales are valid only in the linear-elastic operating range of the model, in which the elastic modulus is constant. Exceeding the limit of linear elasticity leads to changes in the value of the elastic modulus, and, in consequence, the similarity criteria lose their validity. Hence, determining the range of linear-elastic behaviour of the model is extremely important for dynamic tests on a shaking-table.

Generally, dynamic tests on shaking-tables are commonly used to determine either the frequency and mode of natural vibrations of the structure or the dynamic response of the structure to seismic excitations. The results for the prototype are obtained based on the results of the laboratory model and the similarity scales. The shaking-table accelerations, which should be applied to the model, result from the acceleration similarity scale. The values of the acceleration similarity scales of the selected polymers range from 0.21 (for the 30D filament) to 24.33 (for the ULTRA-PLA filament) (see Table 6). In the case of a large value of this scale, large acceleration amplitudes must be executed on a shaking-table. This may result in non-linear model behaviour.

**Figure 9:**
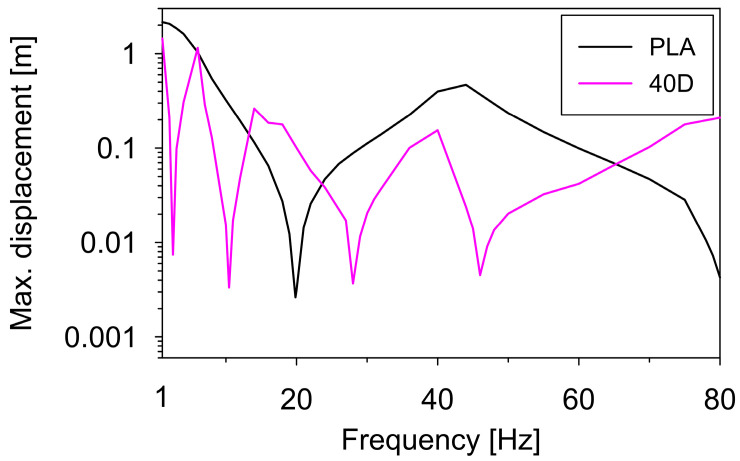
The maximum shaking-table displacements at which the models made of the ULTRA-PLA and the 40D filament operate in the linear-elastic range.

For a natural frequency determination, sweep excitation at a frequency close to the expected natural frequency is usually applied to the model. The model operates strictly in the resonance zone and the linear-elasticity limit may be exceeded. Therefore, it is reasonable to numerically estimate the maximum acceleration (or displacement) of the shaking-table beyond which the non-linear work of a model occurs. To recognize the maximum displacement, numerical calculations were performed for two models made of polymers with extremely different acceleration similarity scales, i.e., the model made of the ULTRA-PLA and the 40D material. Sinusoidal kinematic excitations at frequencies from 0 to 80 Hz (every 0.5 Hz) were used to excite the models. The damping ratio of 1% was adopted as a typical value for 3D-printed materials [32,33]. The calculated maximum shaking-table displacements (as a function of the excitation frequency), at which the models made of the ULTRA-PLA and the 40D filament still operate in the linear-elastic range, are shown in Figure 9. For example, the relationship shows that the maximum displacement at the first resonant frequency is 3 mm for the ULTRA-PLA filament, whereas the relationship for the 40D filament indicates the maximum displacements of 7, 3, 4, and 5 mm at the four subsequent natural frequencies. Exceeding these values leads to changes in the elastic moduli of the filament, and therefore, the similarity criteria are no longer valid. 

Next, the dynamic performance of the models under the seismic excitation was analysed. To recognize whether the models operate within or beyond the linear-elastic limits, numerical calculations of their dynamic response to seismic excitation were carried out using the Time History Analysis (THA) method. The seismic event registered in Hatay Seismic Station (Turkey) [34] was adopted as the kinematic excitation. The magnitude of the shake was 7.8, and the Peak Ground Accelerations in two horizontal directions were 5.85 m/s^2^ and 6.52 m/s^2^. The damping ratio of 1% was assumed. The similarity scales used for calculations, the linear-elastic limit, and the maximum stresses obtained at the base of the model made of the ULTRA-PLA and the 40D filament are collected in Table 11. 

The numerical prediction of the dynamic behaviour of ULTRA-PLA and 40D models proved that both models work in a linear-elastic range; thus, the similarity criteria are not changed, and the results of the seismic tests are reliable. A similar conclusion concerning the linear-elastic limit exceedance can be formulated for all analysed polymers.

### 4.6. Practical Example: The Assessment of Suitability of a PLA-IMPACT Filament for 3D-Printed Laboratory Models for Shaking-Table Tests

An example of a comprehensive study on the dynamic characteristics of a reinforced chimney 120 m high can be found in the work [35]. This work contains the results of an in situ experiment performed on the chimney, the results of the shaking-table test, and numerical research on the laboratory model printed with a PLA-IMPACT filament.

The current study presents the assessment of the choice of the PLA-IMPACT filament for printing the laboratory model in the context of (1) the possibility of determining the expected natural frequencies, (2) checking the linear-elastic behaviour of the model on a shaking-table, and (3) determining the maximum displacements of the shaker during the test.

**Figure 10:**
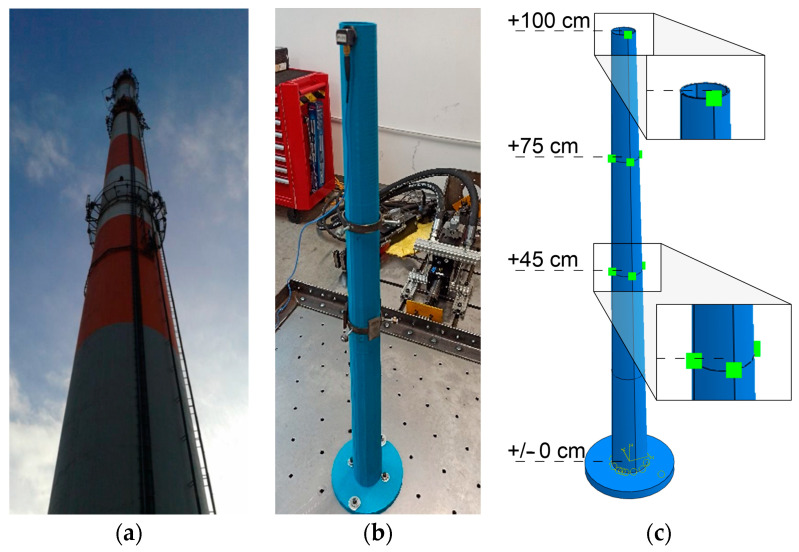
(**a**) The view of the chimney; (**b**) the view of the laboratory model; (**c**) the numerical model of the mock-up [30,35].

The basic natural frequencies of the chimney (see Figure 10a), measured through the in situ experiment, were 0.365 and 1.570 Hz [35]. The dynamic test of the 3D-printed model of the chimney was carried out on a shaking-table, which generated vibrations up to 80 Hz. The geometric similarity scale of 1:120 was implemented, which resulted in the model height of 1 m. The data of the PLA-IMPACT filament are collected in Table 12.

Additional masses, reflecting the ceramic lining in the prototype, were attached to the model at three levels: 1.0, 0.75, and 0.45 m above the ground. According to the mass similarity scale, the values of the masses were 12, 88, and 200 g. At the levels of 0.75 and 0.45 m, the masses were added in the form of steel clamps. At the top level, a 12 g accelerometer was attached to the model. The laboratory model and the numerical model of the mock-up with lumped masses are presented in Figure 10b,c, respectively.

To estimate the approximate ranges for the first and second natural frequencies, the shaking-table generated a horizontal sweep of frequency in the range of 0.2–80 Hz. Then, linear sweeps were performed from 15 to 18 Hz and from 71 to 75 Hz, with a displacement amplitude of 0.2 mm. The natural frequencies obtained through the in situ experiment, the shaking-table tests, and the numerical calculations are summarized in Table 13 [35]. 

**Figure 11:**
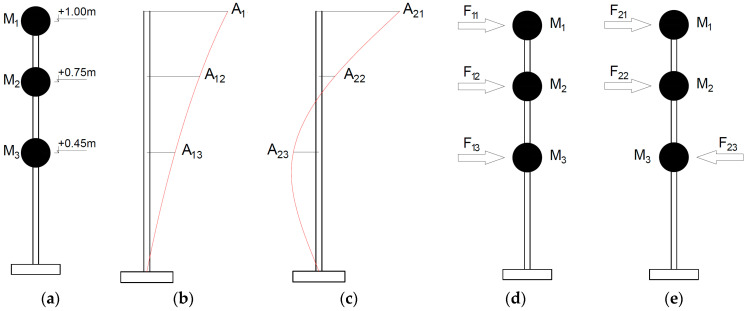
(**a**) The location of the masses; (**b**) the first eigenmode; (**c**) the second eigenmode; (**d**) the inertia forces associated with the first mode; (**e**) the inertia forces associated with the second mode.

In the current study, the stresses in the 3D-printed laboratory model were approximately estimated to assess whether they did not exceed the yield point. The concept of the stresses’ estimation is shown in Figure 11. 

The lumped masses attached to the model were *M*_1_ = 0.100 kg, *M*_2_ = 0.263 kg, and *M_3_* = 0.433 kg. The amplitudes of the first and the second eigenmodes were *A*_11_ = 1.00, *A*_12_ = 0.68, *A*_13_ = 0.34, and *A*_21_ = 1.00, *A*_22_ = 0.16, *A*_23_ = −0.48, respectively. The maximum accelerations recorded at the top of the model while tracking the first and the second frequency were *a*_1_ = 35 m/s^2^ and *a*_2_ = 4 m/s^2^, respectively. Based on these data, the inertia forces acting on the model were calculated for the first and the second natural frequency:

$$F_{11}=M_{1}\cdot a_{1}\cdot A_{11}=3.5\;N,\;F_{12}=M_{2}\cdot a_{1}\cdot A_{12}=6.26\;N,\;F_{13}=M_{3}\cdot a_{1}\cdot A_{13}=5.15\;N$$

$$F_{21}=M_{1}\cdot a_{2}\cdot A_{21}=0.4\;N,\;F_{22}=M_{2}\cdot a_{2}\cdot A_{22}=0.16\;N,\;F_{23}=M_{3}\cdot a_{2}\cdot A_{23}=-0.83\;N$$

Then, the maximum bending moment and maximum stresses at the base of the model under the calculated inertia forces were determined. The maximum stresses in the structure were 0.7 and 0.1 MPa (for the excitation with the first and the second natural frequency), which was much lower than the linear-elastic limit of 36.8 MPa for the PLA-IMPACT filament. Hence, the model operated in the linear-elastic range.

To confirm the linear-elastic operation of the model, the maximum allowable displacements of the shaker were also determined. They were 4 and 7 mm for frequencies in the range of 15–18 Hz and 71–75 Hz (see Figure 9), respectively. It was far above the displacement of 0.2 mm adopted in the experiment.

Based on the above inquiries, some important remarks concerning the choice of the PLA-IMPACT filament for the laboratory model can be formulated. 

Firstly, in the context of determining the natural frequencies, the following aspects are worth noting:

1. Good agreement between the field experiment, the shaking-table test, and the numerical results can be observed; it is generally accepted that the similarity of numerical and experimental natural frequencies is considered satisfactory if the differences are less than 7%.
2. Only two fundamental frequencies can be determined through the tests on the shaking-table with a maximum frequency of 80 Hz, since the frequency similarity scale for the PLA-IMPACT filament takes a large value of 48.69.

Secondly, in the context of the linear-elastic behaviour of the model on a shaking-table, the observations lead to the following conclusions:

1. The linear-elastic limit of the filament was not reached during the research; therefore, the criteria of similarity remained valid.
2. The shaker displacements used to identify the natural frequencies were much smaller than the maximum allowable displacements. It can be predicted that the model behaviour will remain linear-elastic when applying larger displacements during seismic tests.

To sum up, the PLA-IMPACT filament is a good choice for shaking-table models. However, one should be aware of the limitations in identifying natural frequencies due to the large value of the frequency similarity scale.

## 5. Conclusions

A set of seven commercially available polymers has been experimentally tested for their material properties, and the suitability of the polymers for preparing 3D-printed laboratory models for dynamic tests on shaking-tables was assessed. Good agreement between the field experiment, the shaking-table test, and the numerical results were observed. The stresses in the printed 3D model, estimated based on the accelerations registered during the shaking-table test, did not exceed the yield point; thus, the models operated in the linear-elastic range. The numerical prediction on the dynamic performance of the models under strong seismic events has proven that the stresses in the mock-ups were far below the linear-elastic limit, ensuring that the similarity criteria would not change, and the results of the seismic tests would be reliable.

Therefore, laboratory models made of all selected polymers seem to be an economical, effective, and ecological biodegradable option for small-scale shaking-table tests. However, the discussion revealed some issues that ought to be considered before deciding which filament should be used for the 3D printing of a model.

Firstly, the number of model natural frequencies that can be determined is strictly limited by an upper frequency limit of a shaking-table. In the case of large values of frequency similarity scales, the natural frequencies of the model may exceed the upper limit of the shaker excitation frequency. It is strongly indicated to assess the natural frequencies of a laboratory model numerically to avoid underestimating the basic frequencies and to assess the feasibility of their determination.

Secondly, during a natural frequency determination, a model operates strictly in the resonance zone, and the linear-elastic limit may be exceeded. That leads to changes in the elastic moduli of the filament, and therefore, the similarity criteria lose their validity. A practical suggestion to prevent the model from operating beyond the linear-elastic limit is to numerically determine the maximum allowable displacement of the shaking-table prior to testing a model.

## Figures and Tables

**Table 1:** Technological parameters of the samples’ production.

Parameter	Polymer Material						
	PET-G	RGD720	HIPS	ABS	ULTRA-PLA	30D	40D
Extruder temp. [°C]	240	80	250	255	210	240	240
Bed temp. [°C]	90	0	100	110	60	50	50
Speed of extrusion system [mm/s]	200	60	200	200	200	35	35
Layers depth [mm]	0.2	0.016	0.2	0.2	0.2	0.2	0.2
Lamination direction	Cross-hatched (layers +/− 45°)	Longitudinal pattern (layers 0°)	Cross-hatched (layers +/− 45°)	Cross-hatched (layers +/− 45°)	Cross-hatched (layers +/− 45°)	Cross-hatched (layers +/− 45°)	Cross-hatched (layers +/− 45°)
Infill density [%]	99	100	99	99	99	99	99
Form of material	Solid	Liquid	Solid	Solid	Solid	Solid	Solid

**Table 2:** Similarity scales used for the dynamic analyses.

Parameter	Similarity Scale *S*	Equation
Geometry *d*	$\mathrm{Geometry}\;\mathrm{scale}\;S_{d}$	$S_{d}$—dimension base
Elasticity *E*	$\mathrm{Elasticity}\;S_{E}$	$S_{E}$—dimension base
Density *ρ*	$\mathrm{Density}\;\mathrm{scale}\;S_{\rho }$	$S_{\rho }$—dimension base
Time *t*	$\mathrm{Time}\;\mathrm{scale}\;S_{t}$	${S_{t}=S}_{d}\cdot S_{\rho }^{0.5}\cdot S_{E}^{-0.5}$
Frequency *f*	$\mathrm{Frequency}\;\mathrm{scale}\;S_{f}$	$S_{f}=S_{d}^{-1}\cdot S_{\rho }^{-0.5}\cdot S_{E}^{0.5}$
Acceleration *a*	$\mathrm{Acceleration}\;S_{a}$	${S_{a}=S}_{d}^{-1}\cdot S_{\rho }^{-1}\cdot S_{E}$
Mass *m*	$\mathrm{Mass}\;\mathrm{scale}\;S_{m}$	${S_{m}=S}_{d}^{3}\cdot S_{\rho }$

**Table 3:** Elastic moduli obtained from the tensile, the compression, and the bending test with standard deviations, as well as the average values of the elastic moduli of all polymers.

Filament	Tensile Test Results		Compression Test Results		Three-Point Loading Test Results		Average Value of Elastic Modulus from All Tests [GPa]
	Elastic Modulus [GPa]	Standard Deviation [GPa]	Elastic Modulus [GPa]	Standard Deviation [GPa]	Elastic Modulus [GPa]	Standard Deviation [GPa]	
PET-G	1.642	0.068	1.588	0.036	1.664	0.128	1.631
RDG-720	2.222	0.070	2.248	0.023	2.067	0.074	2.179
HIPS	1.846	0.071	1.669	0.102	1.764	0.058	1.760
ABS	2.126	0.066	1.993	0.078	2.119	0.078	2.079
ULTRA-PLA	3.165	0.144	3.084	0.103	3.257	0.063	3.169
40D	0.045	0.005	0.047	0.005	0.051	0.004	0.048
30D	0.026	0.002	0.022	0.003	0.025	0.003	0.024

**Table 4:** The average values of linear-elastic limits of all polymers.

Filament	Average Value of Linear-Elastic Limit [MPa]
PET-G	24.90
RDG 720	31.21
HIPS	18.03
ABS	26.53
ULTRA-PLA	51.70
40D	2.31
30D	1.80

**Table 5:** The average values of mass density of all polymers.

Filament	Average Value of Mass Density [kg/m^3^]	Standard Deviation [kg/m^3^]
PET-G	1205.23	3.07
RDG 720	1161.84	2.10
HIPS	996.44	3.10
ABS	1072.04	2.74
ULTRA-PLA	1221.84	2.67
40D	1101.34	5.32
30D	1056.45	8.03

**Table 6:** Similarity scale obtained based on experimentally determined material parameter.

Similarity Scale	Polymers Material						
	PET-G	RDG 720	HIPS	ABS	ULTRA-PLA	40D	30D
$\mathrm{Geometry}\;S_{d}$	0.0083	0.0083	0.0083	0.0083	0.0083	0.0083	0.0083
$\mathrm{Elasticity}\;S_{E}$	0.0509	0.0680	0.055	0.0649	0.099	0.0015	0.00075
$\mathrm{Density}\;S_{\rho }$	0.482	0.465	0.399	0.429	0.489	0.441	0.423
$\mathrm{Time}\;S_{t}$	0.026	0.022	0.022	0.021	0.019	0.143	0.198
$\mathrm{Frequency}\;S_{f}$	39.02	45.93	44.59	46.71	54.04	7.00	5.06
$\mathrm{Acceleration}\;S_{a}$	12.69	17.58	16.57	18.18	24.33	0.41	0.21
$\mathrm{Mass}\;S_{m}\cdot 10^{-7}$	2.78	2.68	2.30	2.48	2.83	2.55	2.45

**Table 7:** The distribution of the ceramic lining mass of the chimney (the prototype) and the arrangement of the additional masses of the model.

The Prototype		The Model							
Location above the Ground [m]	Additional Mass [Ton]	Location above the Ground [m]	Additional Mass [g]						
			PET-G	RDG-720	HIPS	ABS	ULTRA-PLA	40D	30D
120	152	1.00	42	41	35	38	43	39	37
90	450	0.75	126	121	104	112	127	115	110
60	478	0.50	133	128	110	119	135	122	117
30	510	0.25	142	137	118	126	144	130	125
0	280	0.00	78	75	65	69	79	71	68

**Table 8:** The first natural frequency the models obtained numerically and the first frequency of the chimney (the prototype) calculated based on the similarity scale (scaled frequency).

Filament	Numerical Frequency [Hz]	Scaled Frequency [Hz]	Difference between Numerical and Measured Frequency [%]
PET-G	14.431	0.369	1.19
RDG-720	16.968	0.369	1.21
HIPS	16.463	0.369	1.16
ABS	17.261	0.370	1.24
ULTRA-PLA	19.968	0.370	1.24
40D	2.584	0.369	1.09
30D	1.867	0.369	1.16

**Table 9:** The second natural frequency the models obtained numerically and the second frequency of the chimney (the prototype) calculated based on the similarity scale (scaled frequency).

Filament	Numerical Frequency [Hz]	Scaled Frequency [Hz]	Difference between Numerical and Measured Frequency [%]
PET-G	58.565	1.501	4.41
RDG-720	68.962	1.501	4.37
HIPS	66.849	1.499	4.50
ABS	70.126	1.501	4.37
ULTRA-PLA	81.139	1.502	4.36
40D	10.527	1.503	4.26
30D	7.587	1.500	4.43

**Table 10:** The natural frequencies of the chimney (prototype) and the natural frequencies of models printed with the selected polymers.

	Frequency Similarity Scale	Frequency [Hz]				Modes Available to Detect on Shaking-Table
		I	II	III	IV	
Prototype	1	0.365	1.57	4.40	6.91	
PET-G	39.02	14.24	61.26	171.69	269.63	I–II
RDG 720	45.93	16.76	72.11	202.09	317.38	I–II
HIPS	44.59	16.28	70.01	196.20	308.12	I–II
ABS	46.71	17.05	73.33	205.52	322.77	I–II
ULTRA-PLA	54.04	19.72	84.84	237.78	373.42	I
40D	7.00	2.56	10.99	30.80	48.37	I–IV
30D	5.06	1.85	7.94	22.26	34.96	I–IV

Legend: grey color—Detection impossible.

**Table 11:** Expectation of the linear-elastic limit exceedance.

Filament	Acceleration Similarity Scale [-]	Frequency Similarity Scale [-]	Linear-Elastic Limit [MPa]	Maximum Obtained Stress [MPa]	Exceeding the Linear Limit
ULTRA-PLA	24.33	54.04	51.7	8.7	NO
40D	0.41	7.00	2.31	0.14	NO

**Table 12:** Data for assessment.

		Similarity Scale						
Elastic Modulus [GPa]	Linear-Elastic Limit [MPa]	Density [g/cm^3^]	$\mathrm{Geometry}\;S_{d}$	$\mathrm{Elasticity}\;S_{E}$	$\mathrm{Density}\;S_{\rho }$	$\mathrm{Mass}\;S_{m}$	$\mathrm{Frequency}\;S_{f}$	$\mathrm{Acceleration}\;S_{a}$
2.96	36.8	1.28	0.00833	0.0842	0.512	2.96·10^−7^	48.688	19.754

**Table 13:** The natural frequencies obtained through the in situ experiments, the shaking-table tests, and the numerical calculations.

Frequency Number	Frequency [Hz] Obtained from:			Difference between Shaking-Table and Measured Frequency [%]	Difference between Numerical and Shaking-Table Frequency [%]
	Field Experiment	Shaking-Table Test (Scaled)	Numerical Test (Scaled)		
1	0.365	17.184 (0.35)	17.528 (0.36)	2.77	1.98
2	1.570	73.263 (1.51)	78.242 (1.61)	3.82	6.78
3	-	NA	227.860 (4.68)	-	-
4	-	NA	318.832 (6.55)	-	-

Legend: NA with grey color—detection impossible.

## Data Availability

Data are contained within the article.
